# Cancer stem cell biomarkers in locally advanced head and neck squamous cell carcinoma^☆^

**DOI:** 10.1016/j.bjorl.2025.101689

**Published:** 2025-07-29

**Authors:** Miguel Caballero-Borrego, Juan J. Grau, Neus Basté, Paola C. Castillo, Cristina Teixido, Izaskun Valduvieco, Isabel Vilaseca

**Affiliations:** a Hospital Clinic of Barcelona, Otolaryngology Department, Barcelona, Spain; b Universitat de Barcelona, Facultat de Medicina i Ciències de la Salut, Department of Surgery and Medical-Surgical Specialties, Barcelona, Spain; c Institut d'Investigacions Biomèdiques Agusti Pi Sunyer (IDIBAPS), Barcelona, Spain; d Hospital Clínic, Department of Medical Oncology, Barcelona, Spain; e Hospital Clínic, Department of Pathology, Barcelona, Spain; f Hospital Clinic, Department of Radiation Oncology, Barcelona, Spain; g Head Neck Clinic, AGAUR, Barcelona, Spain

**Keywords:** Head and neck cancer, Biomarkers, HLA-I, CD44, Cancer stem cells

## Abstract

**Objectives:**

To analyze the presence of cancer stem cells markers in consecutive patients with locally advanced Head and Neck Squamous Cell Carcinoma (HNSCC) and establish their suitability as a prognostic biomarker in samples from routine practice.

**Methods:**

Retrospective study of 104 consecutive patients with locally advanced HNSCC treated with definitive surgery and adjuvant chemoradiotherapy. Immunohistochemical expression of CD44, HLA-I, pan-cytokeratin, and phosphorylated Epidermal Growth Factor Receptor (p-EGFR) were evaluated in surgical specimens. Overall Survival (OS) and Disease-Free Survival (DFS) rates were calculated based on clinical characteristics and the total positive score (for tumor marker expression). Hazard ratios were calculated by Cox multivariate analysis.

**Results:**

After a median follow-up of 50-months, higher OS rates were observed for patients without cervical node pathological involvement (p < 0.001), patients with HLA-I overexpression and a total positive score ≥ 15% (p = 0.017), and patients with low CD44 expression and a total positive score ≤ 60% (p = 0.022). Grouping by p-EGFR (p = 0.648) or pan-cytokeratin (p = 0.477) expression did not show statistical differences in OS. None of the biomarkers were associated with different DFS rates. Cox multivariate analysis revealed that positive cervical nodes (HR = 1.294; 95% CI 1.025–1.634; p = 0.030) and the expressions of HLA (HR = 0.373; 95% CI 0.168‒0.829; p = 0.015) and CD44 (HR = 2.170; 95% CI 1.031–4.569; p = 0.041) were independently associated with OS.

**Conclusion:**

Nodal involvement, HLA-I overexpression, and decreased CD44 expression are independent prognostic factors of survival in patients with locally advanced HNSCC.

**Level of evidence:**

III.

## Highlights

• Patients with carcinoma of the head and neck respond differently to treatment.
• The presence of cancer stem cells may explain the tumor resistance to treatment.
• Some cancer stem markers are CD44, HLA-I, pan-cytokeratin, and EGFR.
• HLA-I and CD44 are prognostic factors for survival, but not pan-CK or EGFR.
• Knowing the type of stem cells that integrate a cancer could help guide treatment.

## Introduction

Patients diagnosed with locally advanced Head and Neck Squamous Cell Carcinoma (HNSCC) are generally treated with a multidisciplinary treatment approach,^1^ with the majority undergoing surgery followed by adjuvant platinum-based chemoradiotherapy.^2^ However, despite receiving multidisciplinary treatment with curative intent, more than 50% of patients will relapse within the following three years,^3^ suggesting the existence of biological differences between tumors. The observed differences can be partially attributed to tumor heterogeneity and potential tumor resistance mechanisms associated with treatment.^4^^,^^5^

HNSCC shows cellular heterogeneity, with cells demonstrating the capacity to drive tumor formation in distinct ways.^6^ Based on this observation, some authors have hypothesized that tumors are sustained by Cancer Stem Cells (CSCs). These cells are implicated in tumorigenesis and coexist with non-tumorigenic populations.^4^ These findings were initially observed in hematologic malignancies,^7^ breast cancer,^8^ and neurological malignancies.^9^ CSCs are present in tumors and display characteristics consistent with normal stem cells, including the capacity to generate all cell types associated with a specific cancer. These cells contribute to tumor formation, growth, and metastasis by remaining undifferentiated and self-renewing.^10^^,^^11^ These features regulate select cytoplasmic transcription factors and surface markers that distinguish CSCs from other cells. Previously described markers include Cluster of Differentiation 44 (CD44), HLA class I markers (HLA-I), pan-Cytokeratin (pan-CK), and phosphorylated Epidermal Growth Factor Receptor (p-EGFR).^12^

CD44 is a transmembrane glycoprotein, a receptor for hyaluronic acid, and a co-receptor for growth factors and cytokines.^11^ The CSC subpopulation expressing CD44 demonstrates an increased ability to develop new tumors in a mouse xenograft model of HNSCC.^4^ These findings suggest that CD44 could serve as a cellular marker of CSCs that are potentially resistant to chemotherapy and radiotherapy.^13^ Stemness capacity has been reported in prostate cancer cells without surface differentiation or HLA class I (HLA-I) markers. Tumor cells with low HLA-I expression have been shown an elevated level of resistance to chemotherapy.^14^ The loss of pan-CK expression in the cytoplasm, as determined by immunohistochemical analysis, has been reported to indicate poorly differentiated cells, which is typically observed in tumors with CSC enrichment.^15^ EGFR, overexpressed in 95% of HNSCCs,^16^ has been implicated in chemotherapy resistance and is associated with an increase in CSC-like populations in these tumors, though the mechanism is not fully understood.^17^ Finally, CD44 has been demonstrated to interact with EGFR phosphorylation and activation in vitro in HNSCC cells, and it has been observed that a decrease in CD44 levels is associated with a reduction in p-EGFR and tumor growth.^18^

Stem cell research is becoming increasingly relevant in the context of head and neck cancer by improving prognosis and predicting clinical outcomes. Our group has previously reported enrichment of stem cell expression with the markers CD44, HLA-I, pan-CK, and p-EGFR in relapsed HNSCC after induction chemotherapy compared to the primary tumor.^12^ In this study, we hypothesized that locally advanced HNSCC with a high percentage of cells with pan-CK or HLA-I markers or a low percentage of cells with p-EGFR or CD44 markers would have better clinical outcomes after curative treatment (surgery followed by adjuvant therapy). A comprehensive understanding of the behavior and molecular characteristics of CSCs in otolaryngologic cancers enables the development of more targeted and effective treatments. Stem cell-based biomarkers also improve early detection and therapy response prediction, enabling personalized and adaptive treatment strategies. As research advances, integrating stem cell science into oncologic otolaryngology may significantly enhance survival and long-term quality of life.

## Methods

### Patients

A retrospective cohort study was conducted on consecutive patients with non-metastatic locally advanced HNSCC who underwent definitive surgery plus neck dissection followed by adjuvant chemoradiotherapy, as recommended by a multidisciplinary tumor board at a tertiary referral hospital. No patient was enrolled in clinical trials. All tumors were resectable stage III or IV tumors according to the 7th edition TNM classification.^19^ Only patients with complete surgical resection and no residual microscopic disease were included. Primary tumor and/or nodal tissue samples were available for all patients for pathological evaluation. All patients provided written informed consent, and the study was approved by the institutional ethics and clinical trials committee.

### Adjuvant treatment

Postoperative adjuvant treatment was administered to prevent local recurrence and distant metastasis. The radiation therapy was initiated in the sixth week after surgery, deliverting 55–60 Gy over six weeks. Concurrently, high-dose cisplatin (100 mg/m^2^) was administered intravenously on days 1, 22, and 43. If hearing impairment or renal dysfunction occurred, cisplatin was replaced with carboplatin (Area Under the Curve [AUC = 5]) on the same schedule.

### Pathological and immunohistochemical analysis

All tumor specimens underwent pathological confirmation of squamous cell carcinoma. Blocks from primary tumors or metastatic nodes were selected, stained with hematoxylin and eosin, and analyzed for biomarkers. Each sample was fixed in 10% formaldehyde, paraffin-embedded, and sectioned into ten 5 μm slices mounted on frosted glass slides. Two head and neck pathologists independently reviewed each slide, resolving any disagreements by consensus.

Our institution stores paraffin-embedded tissue blocks under controlled conditions, including limited light exposure, low humidity, and constant room temperature, in a medical archive to ensure long-term preservation and antigen stability for reliable histologic and immunohistochemical analysis for clinical trials and retrospective studies. Some samples are also stored at cooler temperatures in an off-site university biobank for extended research use.

Immunohistochemical staining was performed using the following antibodies: purified mouse anti-human CD44 monoclonal antibody (G44-26); HLA-I monoclonal antibody (ab52922, Abcam, UK); anti-pan-Cytokeratin C-11 antibody for pan-CK (ab7753 MoEF, Abcam, UK); and anti-EGFR antibody for p-EGFR (phospho Y1068, EP774Y, Abcam, UK).

The Total Positive Score (TPS) was defined as the percentage of malignant cells positive for each marker relative to the total number of malignant cells observed under optical microscopy. TPS cut-offs were set at 60% for CD44, 15% for HLA-I, 95% for pan-CK, and 1% for p-EGFR, based on the reference values from a previous study.^12^ Patients were classified into two groups according to their stem cell biomarker expression levels.

### Statistical analysis

Patient characteristics are presented as median and Interquartile Range (IQR) or mean and Standard Deviation (SD) for quantitative variables, or as number and percentage for qualitative variables. Continuous variables were compared using analysis of variance, using qualitative variables using the Chi-Squared or Fisher’s exact test. Multivariate Cox regression was used to identify independent factors associated with survival. The results were reported as Hazard Ratios (HR) and 95% Confidence Intervals (95% CI). Overall Survival (OS) was measured from the date of surgery to death from any cause or last follow-up. Disease-Specific Survival (DSS) included only tumor- or treatment-related deaths. OS and DSS were analyzed using the Kaplan–Meier method and log-rank test.^20^ Multivariate Cox regression was applied to assess factors independently associated with OS and Progression-Free Survival (PFS), with results expressed as HRs and 95% CIs. Statistical analyses were conducted using IBM SPSS, Version 25.0 (IBM Corp., Armonk, NY, USA), with p-values < 0.05 considered significant.

## Results

### Patient and treatment

The study included 104 consecutive patients who met the inclusion criteria. Demographic, clinical, tumor, and treatment characteristics are summarized in Table 1.

**Table 1:** Baseline patient characteristics and description of tumors and treatments.

	Median (IQR)	64.5 (36–96)
Age, years	Under 65; n (%)	52 (50%)
Gender; n (%)	Male	88 (85)
Tobacco abuse; n (%)	Yes	75 (72.1)
Tobacco (pack/year)	Mean (SD)	37.3 (0−110)
Alcohol intake; n (%)	No	55 (52.9)
	Moderate	24 (23.1)
	Severe	25 (24)
Primary location; n (%)	Oropharynx	9 (8.7)
	Larynx	61 (58.7)
	Hypopharynx	4 (3.9)
	Oral cavity	28 (26.9)
	Nasal cavity	2 (1.9)
Grade of differentiation; n (%)	Well differentiated	6 (5.8)
	Moderately differentiated	63 (60.6)
	Poorly differentiated	29 (27.9)
	Undifferentiated	3 (2.9)
	Other	3 (2.9)
T category; n (%)	Tx	1 (1)
	T1	5 (4.8)
	T2	15 (14.4)
	T3	26 (25)
	T4	57 (54.8)
N category; n (%)	N0	41 (39.4)
	N1	27 (26)
	N2a	1 (1)
	N2b	19 (18.3)
	N2c	15 (14.4)
	N3	1 (1)
Cervical nodes; n (%)	Positive	62 (59.6)
	Negative	42 (40.4)
Stage; n (%)	III	25 (24)
	IVa,b	79 (76)
Adjuvant Treatment; n (%)	None	12 (11)
	Radiotherapy only	29 (27.5)
	Chemoradiotherapy	63 (60.5)
Type of chemotherapy; n (%)	Cisplatin	30 (28.8)
	Carboplatin	33 (31.7)
	No chemotherapy	41 (39.4)

### Immunohistochemical results for stem cell markers

**Fig. 1:**
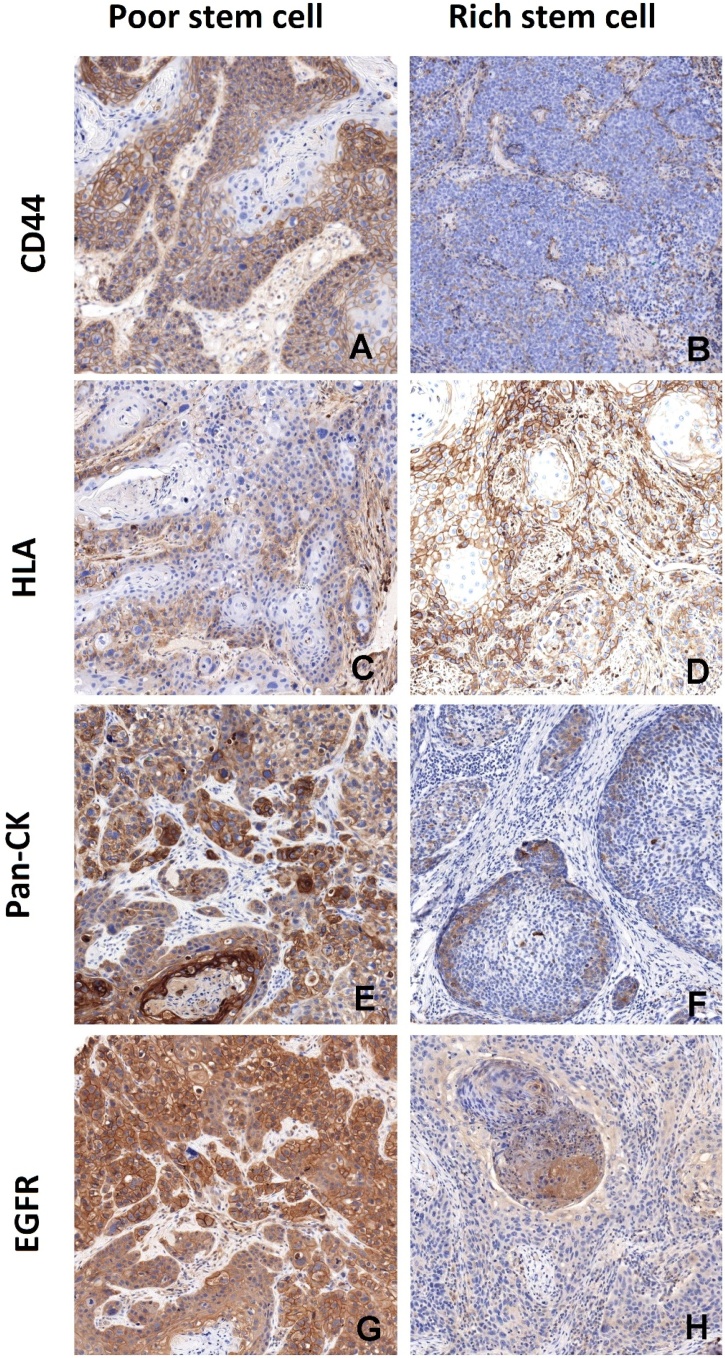
Immunohistochemical characteristics of patient tissues with different prognoses.

The pathological analysis was performed on primary tumor samples in 81 patients (77.8%). In the remaining 23 cases (22.1%), cervical lymph node metastases were analyzed due to insufficient or poorly preserved primary tumor tissue. The results for marker expression were as follows: pan-CK had a TPS of 95%–100% in 76 patients (76.8%); HLA-I had a TPS of ≥ 15% in 44 patients (43.4%); CD44 (high-intensity staining) had a TPS of ≥ 60% in 31 patients (31.3%); and p-EGFR had a TPS of ≥ 1% in 22 patients (21.2%). Overall, 58 patients (58.6%) expressed more than one stem cell biomarker. Fig. 1 illustrates the immunohistochemical features of tumors with stem cell enrichment.

### Survival analysis

The median survival at follow-up was 50 months (IQR, 16–87 months). Table 2 shows the results of the univariate analysis for the relationship between patient and tumor characteristics and the type of treatment with tumor-related mortality. The probability of tumor-related mortality was found to be significantly associated with nodal status (p < 0.001), HLA expression (p = 0.0034), and the expression of several CSC biomarkers (p = 0.008).

**Table 2:** Association of patient and tumor characteristics and treatment type with mortality.

Variable	Tumor-related death (%)	p-value
Age (≤65 / >65)	36.7 / 38	0.896
Gender (Female / Male)	26.7 / 39.3	0.352
Smoking habit (No / Yes)	48.1 / 33.3	0.175
Alcohol intake (No / Yes)	36.5 / 38.3	0.857
Pan-CK (No / Yes)	21.7 / 42.1	0.077
HLA-1 (No / Yes)	46.4 / 25.6	0.034
CD44 (No / Yes)	33.8 / 45.2	0.280
>1 CSCs biomarker (No / Yes)	22 / 48.3	0.008
EGFR (No / Yes)	38.5 / 33.3	0.666
Grade of differentiation (well-moderate / poor-undifferentiated)	40.3 / 32.3	0.445
T (Tx‒T2 / T3‒T4)	47.4 / 35	0.316
Stage (III / IV)	33.3 / 38.5	0.666
Location (OC-OP-SF /HP-L)	41.2 /35.4	0.572
Nodal stage (Negative / Positive)	15.4 / 51.7	<0.001
Chemotherapy (No / Yes)	38.5 /36.7	0.857
Type of chemotherapy (Carboplatin / Cisplatin)	36.4 / 37.4	0.957

CSCs, Cancer Stem Cells; HP, Hypopharynx; L, Larynx; OC, Oral Cavity; OP, Oropharynx; SN, Sinonasal.

**Fig. 2:**
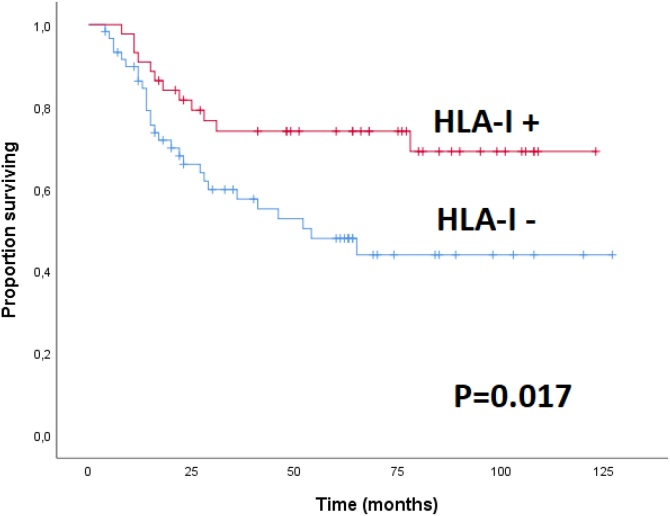
Overall survival by HLA-I expression HLA+, tumor expression of HLA-I ≥15%; HLA-, tumor expression of HLA-I < 15%.

**Fig. 3:**
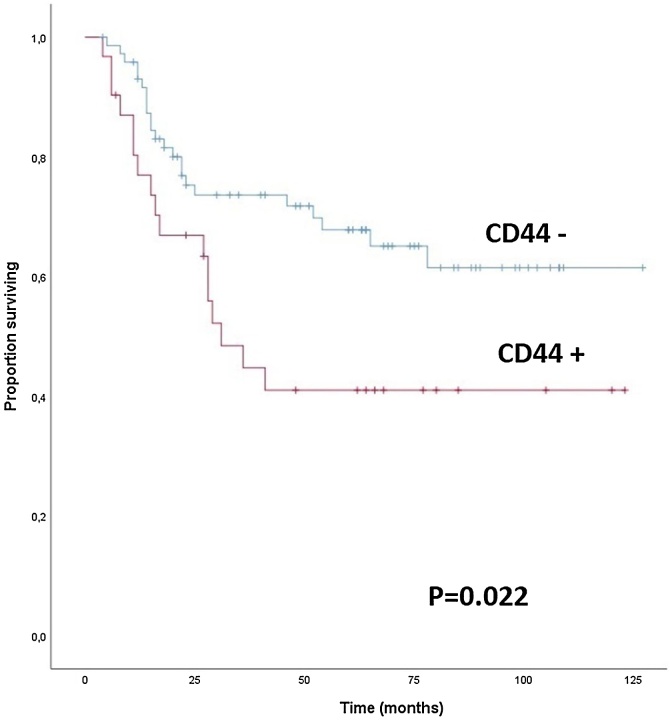
Overall survival by CD44 expression CD44+, tumor expression of CD44 ≥ 60%; CD44-, tumor expression of CD44 < 60%.

OS and DSS showed no significant differences based on age, gender, differentiation, location (oral cavity-oropharynx vs. larynx-hypopharynx), stage (III vs. IV), tumor size (Tx‒T2 vs. T3‒T4), adjuvant treatment, or type of chemotherapy (cisplatin vs. carboplatin). OS was significant better in patients with high HLA-I expression (p = 0.017, Fig. 2) or low CD44 expression (p = 0.022, Fig. 3), but not in those with p-EGFR (p = 0.648) or pan-CK (p = 0.477) expression. DSS did not differ significantly among the groups.

Cox proportional hazards regression was used to assess patient and tumor characteristics, biomarker expressions, and treatments. Positive cervical nodes (HR = 1.294; 95% CI 1.025–1.634; p = 0.030) and expressions of HLA (HR = 0.373; 95% CI 0.168–0.829; p = 0.015) and CD44 (HR = 2.170; 95% CI 1.031–4.569; p = 0.041) were identified as independent factors associated with OS.

## Discussion

Few studies have assessed the expression of several CSC biomarkers in surgical specimens from patients with primary locally advanced HNSCC showing positive results for their use as prognostic factors. Variability in patient response and prognosis following a standard treatment protocol suggests that these tumors, though all squamous cell carcinomas, are biologically heterogeneous. This heterogeneity may be related to differences in the CSC populations influencing treatment response.^21^ Therefore, this study evaluated the expression of CSCs based on four biomarkers characteristic of these cells: pan-CK, CD44, p-EGFR, and HLA-I. Our findings reveal a strong association between survival and both decreased CD44 and increased HLA-I expression, suggesting that these biomarkers may serve as independent prognostic factors.

HNSCC is characterized by high cytokeratin expression. Pan-CK is a broad-spectrum epithelial marker that is routinely used in diagnostic pathology to confirm the epithelial origin of tumor cells. This is particularly valuable in complex tissues like HNSCC, where tumor boundaries may be obscured by inflammatory or stromal components.^22^ The inclusion of pan-CK in our study was supported by experimental evidence from both animal and human studies, where pan-cytokeratin, alone or in combination with other markers, has been linked to stem and progenitor cells populations in both normal and pathological tissues. In mouse models, pan-CK is expressed in the basal and intermediate layers of the mucosa, suggesting a role in cell differentiation.^23^ Since the expression of pan-CK is reduced in stem cells, we can assume that the expression of this differentiation protein is also reduced in CSCs.^24^ Grau et al. demonstrated that relapsing HNSCC tumors showed a decrease in the percentage in the neoplastic cells positive for cytoplasmic pan-CK and positive for other stem cell biomarkers. This suggests tumor enrichment in stem cells and poorer prognosis.^12^ The inclusion of pan-CK in our study was supported by experimental evidence from both animal and human studies, where pan-cytokeratin, alone or in combination with other markers, has been linked to stem and progenitor cells populations in both normal and pathological tissues.

CD44 is a type I transmembrane glycoprotein that is expressed in the cancer cell surface membrane and is essential for cellular adhesion, migration, and proliferation.^25^ CD44 can even be detected in the saliva of patients with precancerous lesions or oral tumors.^26^ The role of CD44 as a CSC marker in HNSCC has been the subject of extensive research, showing associations with tumor growth, metastasis and chemo-radiotherapy resistance. This correlation holds implications for diagnosis, prognosis, and therapeutic targeting.^27^^,^^28^ In the present study, CD44 overexpression was identified as a factor independently associated with poorer survival. Other authors have also identified a correlation between high levels of CD44 expression and various adverse outcomes in patients with HNSCC, including cancer recurrence,^29^^,^^30^ poor response to radiotherapy,^31^ regional metastasis,^32^ and reduced overall survival.^10^^,^^11^^,^^33^^,^^34^ Joshua et al.^35^ demonstrated that patients diagnosed with HNSCC who escape currently available therapies had a higher percentage of CD44+ cells. Kavitha et al.^34^ found that CD44 mRNA transcript and protein expression levels were significantly higher in early primary HNSCC tissues than in normal tissues, and high CD44 expression was correlated with poor survival. It was also noted that the overexpression of CD44 is more pronounced in HPV-negative tumors and those with TP53 mutations in HNSCC.^34^ Dubey et al.^36^ discovered that the expression of CD44 is associated with resistance to radiotherapy and poor survival outcomes. One of the most interesting articles addressing this topic in oral squamous cell carcinoma is a meta-analysis presented by Mirhashemi et al.^28^ published in 2023. This study demonstrated that high CD44 expression is associated with metastasis to lymph nodes and distant metastasis, poorer survival of the patients, tumor recurrence, higher tumor stage and grade and aggressive clinicopathological features. In conclusion, CD44 can serve as a potential diagnostic and prognostic biomarker for HNSCC^34^ and oral cancer,^28^ offering new molecular targets for CD44-targeted therapy for cancer management.

EGFR plays a key role in the maintenance of epithelial tissues. However, when EGFR signaling is altered, it becomes the grand orchestrator of epithelial transformation.^37^ EGFR is frequently expressed at elevated levels in various forms of cancer and its expression frequently correlates positively with cancer progression and poor prognosis.^38^^,^^39^ EGFR-targeted therapies have led to a the new era of precision-oncology.^37^ EGFR is believed to be activated to p-EGFR, and it may be associated with tumor staging or grading.^40^ Some studies have demonstrated that p-EGFR is a prognostic biomarker.^41^, ^42^, ^43^, ^44^ However, in a study of patients with HNSCC, the expression of EGFR did not correlate with EGFR autophosphorylation.^45^ These findings suggest tumor heterogeneity with a weak association between global EGFR expression and p-EGFR. In contrast, elevated p-EGFR expression has been reported to correlate with lymph node metastasis in patients with adenoid cystic salivary gland tumors, supporting the hypothesis that elevated p-EGFR expression facilitates lymphatic metastasis.^46^ Moreover, p-EGFR in lung cancer cells has been identified as a potent CSC biomarker, associated with a strong sensitivity to anti-EGFR agents (e.g., erlotinib) in vitro and in vivo.^47^ In a panel of HNSCC cell lines, p-EGFR levels have also been associated with a high response to cetuximab, a monoclonal anti-EGFR antibody, but not with cisplatin therapy.^48^ Studies of nasopharyngeal carcinoma have further demonstrated a correlation between high EGFR^49^ expression and poor OS, but not with p-EGFR.^50^ In our series, p-EGFR expression in tumor specimens and cervical nodes was comparable, with a TPS of ≥1% in 21% of patients. However, we observed no significant differences in OS or DSS by p-EGFR expression, suggesting that p-EGFR is not a suitable cancer CSC biomarker in these patients. However, the variability in our results and that of a large number of studies could be due to the complexity of the EGFR pathway. While our study did not ascertain the suitability of p-EGFR as a stem cell biomarker, there is ongoing research exploring treatments that target the EGFR pathway.^51^^,^^52^

HLA-I overexpression has been associated with favorable clinical outcomes in certain tumors and has gained importance in the new era of “immune checkpoint antibodies”.^53^, ^54^, ^55^ These findings have been observed across different types of head and neck cancers. Mints et al.^56^ studied a cohort of patients with hypopharyngeal squamous cell carcinoma and found that HLA-1 expression was correlated with survival in HPV-negative tumors. The enrichment of the tumor inflammation signature in these patients’ tumors suggests a potential benefit from immunotherapy. They proposed that a low expression of HLA-I could be used to identify patients who may benefit from localized surgery.^56^ Nasman et al.^57^ found that favorable clinical outcome has also been associated with both human papillomavirus-positive and -negative tonsillar squamous cell carcinoma treated with definitive radiotherapy. Ogino et al.^58^ demonstrated a similar association in laryngeal squamous cell carcinoma, suggesting that the reduced extent of CD8 + T-cell infiltration in these tumors may be an important factor.^59^ Wang et al.^60^ conducted a prognostic meta-analysis in nasopharyngeal carcinoma patients and identified a worse response to anti-PD1 and anti-CTLA4 treatment, as well as chemotherapy, in a subgroup characterized by stromal activation, low HLA family expression, and immune checkpoints. Robbins et al.^61^ found that HLA-I expression correlates with the response to neoadjuvant immunotherapy in primary untreated HNC. They also observed that this response is associated with increased tumor cell HLA class I expression after neoadjuvant immunotherapy compared to before treatment. Our results confirm the prognostic significance of HLA-I expression, enhancing its clinical utility for patients with locally advanced stage III or IV HNSCC, regardless of tumor site. Immune activity has been shown to be related to survival in certain head and neck cancers, and enrichment of the tumor inflammation signature suggests a potential benefit from immunotherapy.^56^ The growing understanding of both the antitumor immune response in solid tumors and the role of the immune checkpoint molecules and other immune regulators has led to the development of novel therapeutic strategies that are revolutionizing the clinical management of HNSCC.^62^

Our study has both limitations and strengths. First, we included consecutive unselected patients from tertiary referral center, who had varying comorbidities and exposure risks that may, in part, interfere with standard treatment plans. However, this diversity reflects a real-life scenario and enhances the study’s external validity. Second, to ensure a homogenous sample, we exclusively included non-metastatic locally advanced stage III and IV HNSCC cases with pathologically negative surgical margins. The inclusion of patients treated with upfront surgery improved the quality and sufficiency of tumor tissue for biomarker analysis. Third, the retrospective nature of the study and the limited sample size prevented us from stratifying by tumor site, which would have been of great interest. Finally, the extended recruitment period may have introduced variations in treatment protocols and follow-up.

## Conclusion

Our study demonstrates tumor heterogeneity in the expression of CSC markers in a series of consecutive patients with locally advanced HNSCC treated with surgery and adjuvant chemoradiotherapy. The absence of nodal involvement, overexpression of HLA-I, and decreased expression of CD44 were identified as independent prognostic factors for survival. As an immediate clinical application, routine analysis of these biomarkers could help identify surgical patients in need of novel adjuvant treatments. As a future prospect of the findings, understanding the role of the immune checkpoint molecules and other immune regulators will facilitate the development of innovative therapeutic strategies that have the potential to revolutionize the clinical management of HNSCC.

## CRediT authorship contribution statement

All the authors have made substantial contributions following the rules of the International Committee of Medical Journal Editors (ICMJE).

## Consent for publication

Not applicable (the manuscript does not contain data from any individual person).

## Ethics approval and consent to participate

This study was approved by the Ethics Committee of Hospital Clinic of Barcelona (HCB/2014/0294). All procedures were conducted following the ethical standards of the institutional and national research committee and the tenets of the 1964 Helsinki Declaration and its later amendments. This study is reported according to the STROBE guidelines. As this was a retrospective study, patients’ clinical data were collected without any interference with their treatment and with no risk to the patients’ physiology. The requirement for informed consent was waived and the collected data were protected from disclosure.

## Funding

The authors report no financial support or interests.

## Availability of data and materials

The datasets generated and/or analyzed during the current study are not publicly available due to our institutional protocols, but they will be made available from the corresponding author on reasonable request.

## Declaration of competing interest

The authors declare no conflicts of interest.
